# Optimal treatment strategies for clinically suspicious lateral pelvic lymph node metastasis in rectal cancer

**DOI:** 10.18632/oncotarget.20121

**Published:** 2017-08-10

**Authors:** Hye Jin Kim, Gyu-Seog Choi, Jun Seok Park, Soo Yeun Park, Seung Hyun Cho, Soo Jung Lee, Byung Woog Kang, Jong Gwang Kim

**Affiliations:** ^1^ Colorectal Cancer Center, Kyungpook National University Medical Center, School of Medicine, Kyungpook National University, Daegu, Korea; ^2^ Department of Radiology, Kyungpook National University Medical Center, School of Medicine, Kyungpook National University, Daegu, Korea; ^3^ Department of Oncology/Hematology, Kyungpook National University Medical Center, School of Medicine, Kyungpook National University, Daegu, Korea

**Keywords:** lateral pelvic lymph node dissection, locally advanced rectal cancer, preoperative chemoradiation

## Abstract

**Background:**

Although lateral pelvic lymph node (LPN) metastasis is a major cause of local recurrence in patients with rectal cancer, controversy still remains on the treatment of suspected LPN metastasis, “suspicious LPN”. We aimed to determine the optimal treatment strategies for suspicious LPN, in patients with locally advanced rectal cancer who underwent preoperative chemoradiotherapy (CRT).

**Materials and Methods:**

Of 377 patients who received preoperative CRT for rectal cancer between 2006 and 2013, 84 (22.3%) had suspicious LPNs on pretreatment MRI. Patients’ characteristics, MRI findings, operative and pathologic findings, and oncologic outcomes were analyzed retrospectively.

**Results:**

Of 84 patients with suspicious LPNs, 61 showed good response to CRT on posttreatment MRI (short-axis LPN diameter < 5 mm). Among them, 31 patients underwent TME alone (group A), and 30 underwent TME plus LPND (group B). The remaining 23 patients had persistently suspicious LPNs on post-CRT MRI and underwent TME plus LPND (group C). Pathologic LPN metastasis was confirmed in five patients (16.7%) in group B and 15 (62.5%) in group C. Local recurrence developed in 7 (22.6%), 0 (0%), and 4 (17.4%) patients in groups A, B, and C, respectively. Five patients (16.1%) in group A developed *in situ* LPN recurrences. The 3-year disease-free survival rates were 53.7%, 74.2%, and 46.9% in groups A, B, and C, respectively.

**Conclusions:**

Study findings suggested that LPND cannot be omitted for patients with suspicious LPNs on pretreatment MRI even with good response to CRT. Findings from pretreatment MRI should be considered to determine whether LPND is indicated.

## INTRODUCTION

Preoperative chemoradiotherapy (CRT), followed by total mesorectal excision (TME) is a standard treatment of locally advanced rectal cancer because it reduces local recurrence rate to less than 10% [1–3]. However, some patients with rectal cancer are suspected to have concurrent lymph node metastasis in the pelvic side-wall beyond the TME plane on imaging studies. Although lateral pelvic lymph node (LPN) metastasis in rectal cancer has long been revealed, treatments for these patients have not been established.

Recently, selective lateral pelvic lymph node dissection (LPND) for suspected LPNs metastasis, “suspicious LPNs”, has been suggested in patients with rectal cancer who have undergone preoperative CRT [4–6]. This suggestion is supported by following reasons. Routine LPND has been associated with a higher morbidity rate, but there is a lack of supporting oncologic evidence [7–9]. However, the lateral pelvic side-wall is considered as a major site of locoregional recurrence, and LPND for suspicious LPNs resulted in a high metastatic rate, up to 66% even after CRT [4, 10, 11].

Preoperative CRT may lead tumor downstaging, and it could also sterilize lymph nodes located in mesorectum and lateral pelvic side-wall [2, 12]. Currently, pelvic MRI is generally used to evaluate lymph node metastasis after CRT; however, radiation might shrink lymph nodes, and it could adversely affect the accuracy of radiologic restaging. Subsequently, a recent histological study found that after CRT, 95% of all nodes were ≤ 5 mm and 50% of the metastatic nodes were ≤ 3 mm [13], which suggested that nodal size and morphology assessments might be limited to diagnose mesorectal or LPN metastasis in restaging MRI.

Few studies have evaluated the treatment strategies in patients with suspicious LPNs on initial imaging studies. In the present study, we compared the oncologic outcomes between TME alone and TME plus LPND, based on the degree of response to CRT in LPNs on posttreatment MRI, in rectal cancer patients with clinically suspicious LPNs who underwent preoperative CRT.

## RESULTS

Of 377 patients who underwent preoperative CRT with TME, 84 patients had suspicious LPNs on pretreatment imaging. Clinicopathological characteristics of the patients in the clinically suspicious and negative LPN groups are listed in Table 1. The majority of tumors were located in the lower rectum, although they were more frequently found in the suspicious LPN group than in the negative group (75% vs. 62.5%; *P* = 0.038).

**Table 1:** Patient and tumor characteristics

	Suspicious LPN	Negative LPN	*P* value
	(*n* = 84)	(*n* = 293)	
Age			0.419
≥ 70 years	12 (14.3)	55 (18.8)	
< 70 years	72 (85.7)	238 (81.2)	
Gender			0.895
Male	56 (66.7)	199 (67.9)	
Female	28 (33.3)	94 (32.1)	
Tumor distance from anal verge (cm)			0.038
≥ 5 cm	21 (25.0)	110 (37.5)	
< 5 cm	63 (75.0)	183 (62.5)	
Clinical T stage			0.286
T2	6 (7.1)	35 (11.9)	
T3	64 (76.2)	223 (76.1)	
T4	14 (16.7)	35 (11.9)	
Histologic type			0.178
Well/moderate	74 (88.1)	272 (92.8)	
Poor/mucinous/signet	10 (11.9)	21 (7.2)	
CEA (ng/ml)			0.378
≥ 5	22 (26.5)	63 (22.0)	
< 5	61 (73.5)	224 (78.0)	
Type of Surgery			0.423
Low anterior resection	73 (86.9)	264 (78.3)	
Abdominoperineal resection	11 (13.1)	29 (9.9)	
ypT stage			0.171
ypT0	10 (11.9)	55 (18.8)	
ypT1	2 (2.4)	9 (3.1)	
ypT2	21 (25.0)	50 (17.1)	
ypT3	45 (53.6)	169 (57.7)	
ypT4	6 (7.1)	10 (3.4)	
ypN stage			0.080
ypN0	53 (63.1)	221 (75.4)	
ypN1	20 (23.8)	48 (16.4)	
ypN2	11 (13.1)	24 (8.2)	
Circumferential resection margin			0.461
Positive (≤ 1 mm)	7 (8.3)	18 (6.1)	
Negative (> 1mm)	77 (91.7)	275 (93.9)	
Adjuvant chemotherapy			0.796
Yes	80 (95.2)	274 (93.5)	
No	4 (4.8)	19 (6.5)	

The median follow-up period was 32.3 months. Patients with suspicious LPNs had inferior 3-year DFS rates than those in the negative group (54.3% vs. 74.2%; *P* = 0.002). In detail, the 3-year local recurrence rate in the suspicious group was significantly higher than that in the negative group (13.5% vs. 2.8%; *P* < 0.001), whereas the 3-year distant recurrence-free survival rates were not statistically different between the groups (suspicious vs. negative group, 64.5% vs. 76.3%; *P* = 0.103).

**Figure 1:**
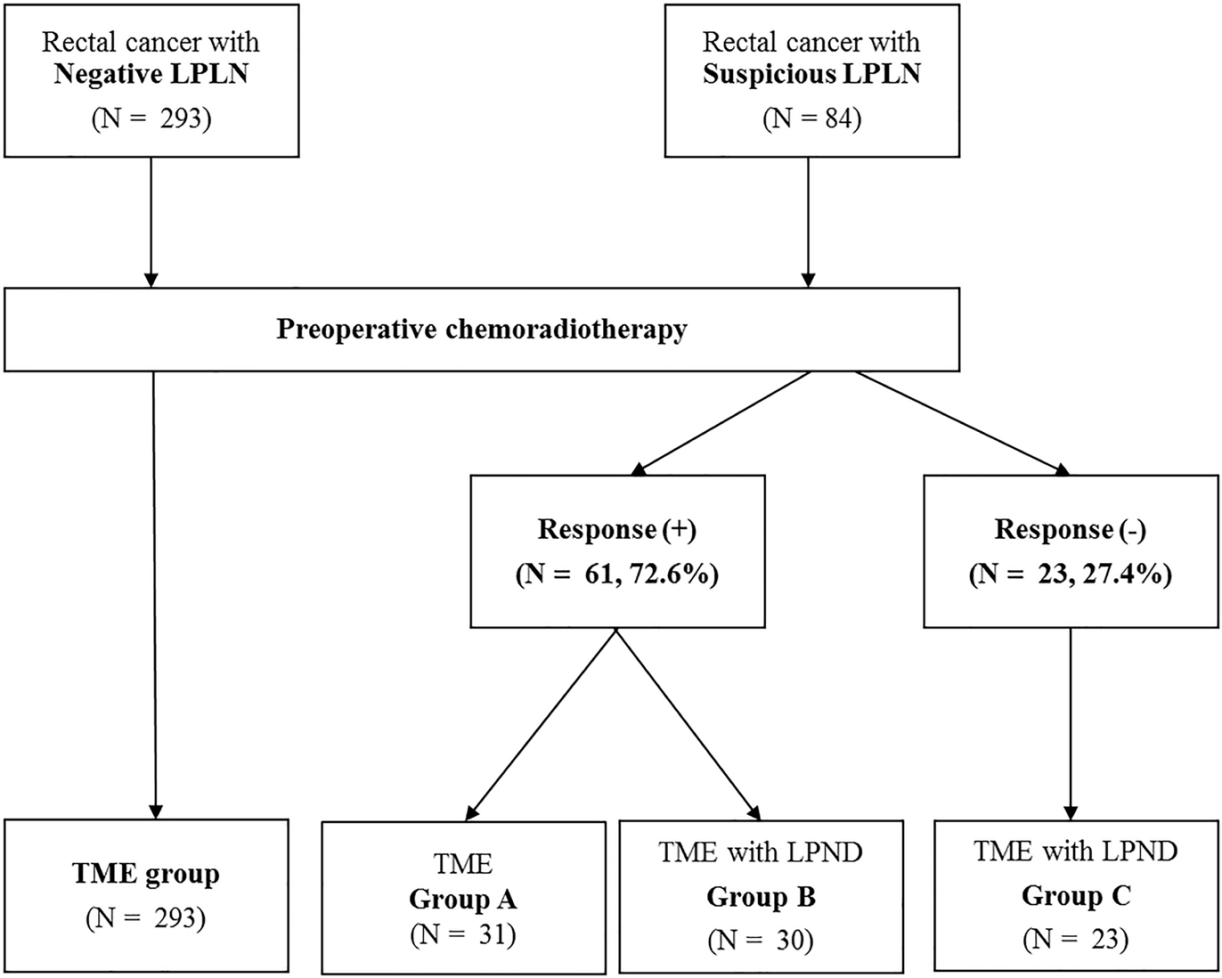
Study patients

Of 84 patients with suspicious LPNs, 61 (72.6%) showed responded pelvic nodes (short-axis diameter < 5 mm) on post-CRT MRI. Among them, 31 patients underwent TME alone (group A) and 30 underwent TME plus additional LPND (group B). The remaining 23 patients (27.4%) did not show an appreciable response to preoperative CRT and had persistently suspicious LPNs on post-CRT MRI. Consequently, they underwent TME with LPND (group C) (Figure 1).

The clinical characteristics of the patients in the three groups are listed in Table 2. There were no significant differences in age, sex, tumor height, clinical T stage, and CEA level among the three groups. The mean short-axis diameters of the LPNs in groups A, B, and C were 7.4, 7.5, and 12.3 mm, respectively, and a short-axis diameter ≥ 7 mm was more frequently observed in group C than in the other groups (*P* = 0.007). Hot-uptake of LPN on pretreatment PET/CT scan was more frequently observed in group C than in the other groups (*P* = 0.014). After preoperative CRT, the mean short-axis diameters were 2.1, 3.5, and 8.9 mm, in group A, B, and C, respectively. Twenty-one patients in group B and 19 in group C underwent bilateral LPND. On pathological examination, ypT3 or T4 was observed more frequently in group C than in the other groups, but the difference was not significant (*P* = 0.119). In addition, ypN positivity was observed more frequently in group C than in the other groups (*P* < 0.001). Five patients (16.7%) in group B and 15 (65.2%) in group C had confirmed LPN metastasis; among them, 3 in group B and 5 in group C had LPN metastasis without mesorectal lymph node metastasis.

**Table 2:** Patient and tumor characteristics among 3 groups

	Group A	Group B	Group C	*P* value
	(*n* = 31)	(*n* = 30)	(*n* = 23)	
Age				0.699
≥ 70 years	5 (16.1)	3 (10.0)	4 (17.4)	
< 70 years	26 (36.1)	27 (90.0)	19 (82.6)	
Gender				0.076
Male	25 (80.6)	16 (53.3)	15 (65.2)	
Female	6 (19.4)	14 (46.7)	8 (34.8)	
Tumor distance from anal verge (cm)				0.164
≥ 5 cm	11 (35.5)	7 (23.3)	3 (13.0)	
< 5 cm	20 (64.5)	23 (76.7)	20 (87.0)	
Clinical T stage				0.714
T2 / T3	26 (83.9)	26 (86.7)	18 (78.3)	
T4	5 (16.1)	4 (13.3)	5 (21.7)	
Histologic type				0.119
Well/moderate	27 (87.1)	29 (96.7)	18 (78.3)	
Poor/mucinous/signet	4 (12.9)	1 (3.3)	5 (21.7)	
CEA (ng/ml)				0.093
≥ 5	9 (30.0)	4 (13.3)	9 (39.1)	
< 5	21 (70.0)	26 (86.7)	14 (60.9)	
Type of Surgery				0.423
Low anterior resection	28 (90.2)	26 (86.7)	19 (82.6)	
Abdominoperineal resection	3 (9.8)	4 (13.3)	4 (17.4)	
Pre-treatment LPLN size				0.007
≥ 7 mm	18 (58.1)	19 (63.3)	22 (95.7)	
< 7 mm	13 (41.9)	11 (36.7)	1 (4.3)	
Pre-treatment PET/CT				0.014
Uptake (+)	14 (53.8)	22 (75.9)	20 (90.9)	
Uptake (−)	12 (46.2)	7 (24.1)	2 (9.1)	
Post-treatment LPLN size				< 0.001
≥ 5 mm	0	1 (3.3)	23 (100)	
< 5 mm	31 (100)	29 (96.7)	0	
LPND				0.232
Unilateral	-	9 (30.0)	4 (17.4)	
Bilateral	-	21 (70.0)	19 (82.6)	
ypT stage				0.119
T0–T2	15 (48.4)	13 (43.3)	5 (21.7)	
T3–T4	16 (51.6)	17 (56.7)	18 (78.3)	
ypN stage				< 0.001
N0	24 (77.4)	24 (80.0)	5 (21.7)	
N1–N2	7 (22.6)	6 (20.0)	18 (78.3)	
Pathologic LPLN metastasis		5 (16.7)	15 (65.2)	< 0.001
Without mesorectal LN metastasis		3 (60.0)	5 (33.3)	
With mesorectal LN metastasis		2 (40.0)	10 (66.7)	

**Figure 2:**
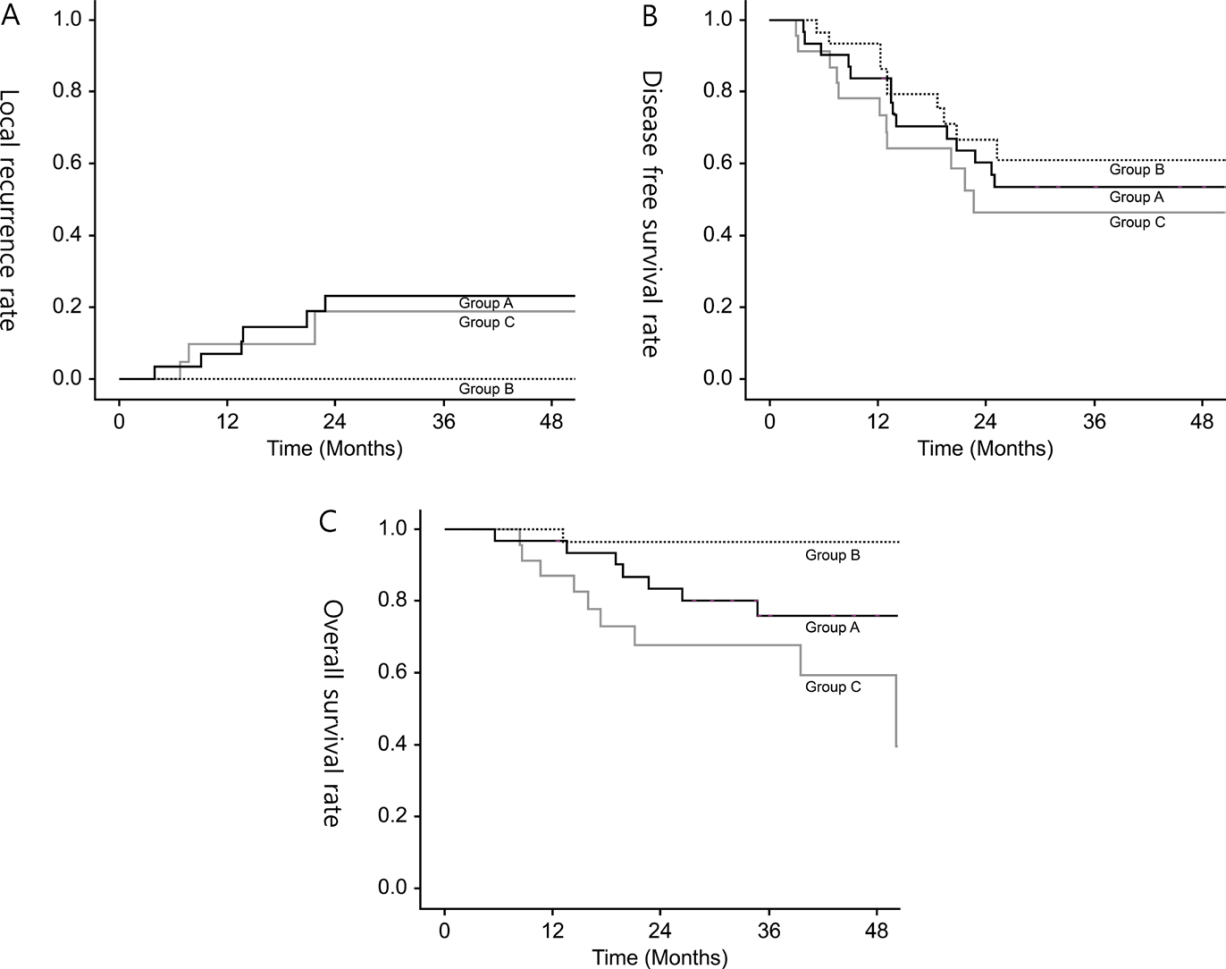
(**A**) 3-year local recurrence rate, (**B**) 3-year disease-free survival rate, (**C**) 3-year overall survival rate in all groups.

The median follow-up period for all patients was 34.1 months (range, 9–70 months). Local recurrence rates at 3 years were significantly greater in groups A and C than in group B (group A vs. C vs. B, 23.1% vs. 18.8% vs. 0%, respectively; *P* = 0.001). The 3-year distant recurrence-free survival rate was lower in group C than those in groups A and B, but the difference was not significant (group C vs. A vs. B, 55.5% vs. 74.3% vs. 61.6%, respectively; *P* = 0.211). The 3-year DFS rates were lower in groups A and C compared than those in group B (group A vs. C vs. B, 53.7% vs. 46.9% vs. 66.1%, respectively; *P* = 0.035). The 3-year OS rate was lower in group C than those in groups A and B, but the difference was not significant (group C vs. A vs. B, 66.7% vs. 80.1% vs. 96.6%, respectively; *P* = 0.327) (Figure 2).

In group A, 11 (35.5%) of 31 patients developed recurrence; among them, 7 (22.6%) experienced local recurrence, and 6 (19.4%) of whom had recurrence in the lateral pelvic side-wall. Five patients (16.1%) developed isolated pelvic node recurrence in the same site, *in situ* LPN recurrence, where LPN metastasis was suspected on pretreatment imaging. Univariate analysis revealed that short-axis diameter ≥ 7 mm on pelvic MRI and hot-uptake on PET/CT scan were significantly associated with local recurrence. All patients who developed local recurrence initially had both risk factors. None of the patients in group B developed local recurrence. In group C, 5 (21.7%) of 23 patients developed local recurrence: Of these, 3 (13.0%) experienced central pelvic recurrences including anastomosis or presacral area and 2 (8.7%) experienced pelvic-side wall recurrences.

**Figure 3:**
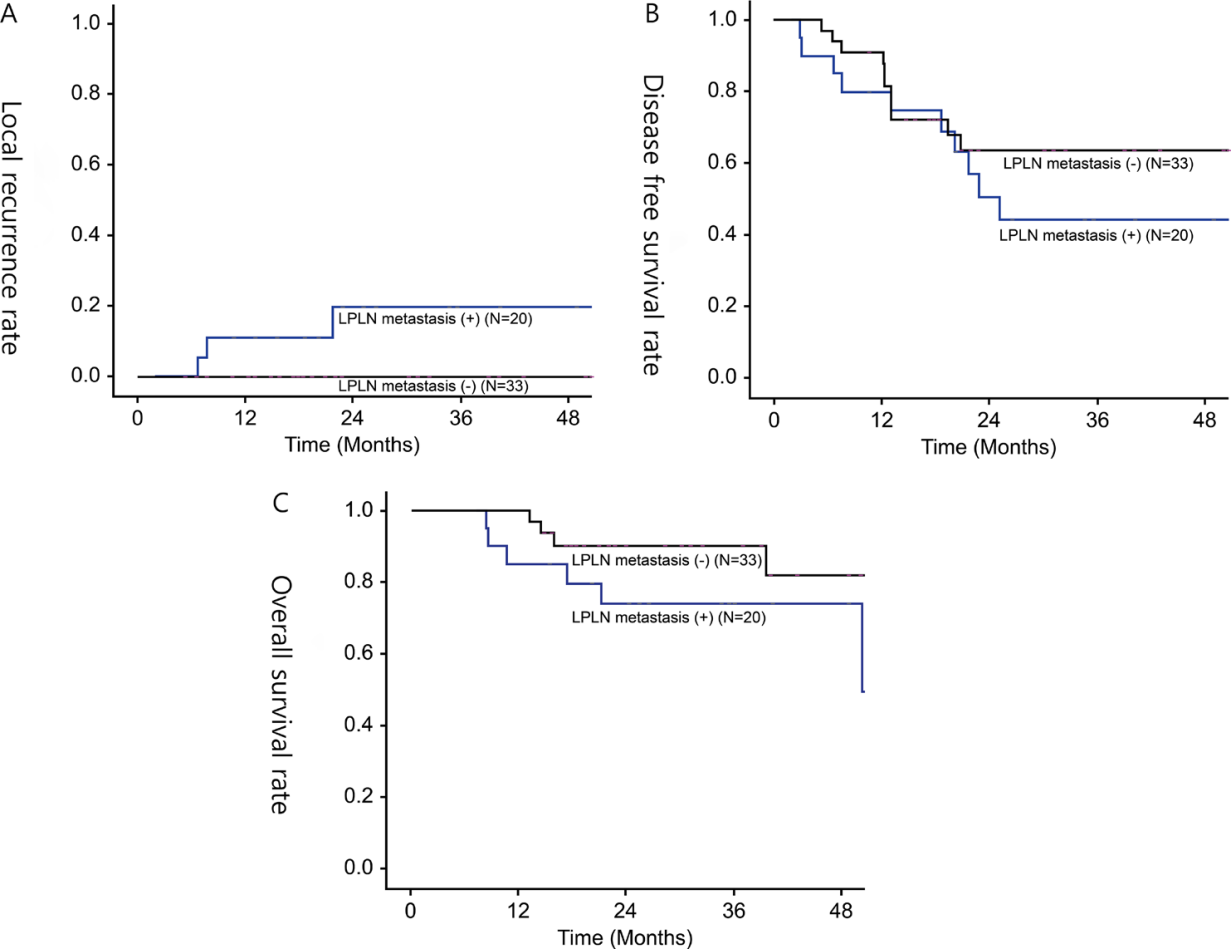
(**A**) 3-year local recurrence rate, (**B**) 3-year disease-free survival rate, (**C**) 3-year overall survival rate in patients with LPN metastasis or without LPN metastasis.

Pathological examination revealed that 20 patients (37.7%) in groups B and C were identified to have LPN metastasis. Patients with LPN metastasis had significantly higher local recurrence rates than those without LPN metastasis (20% vs. 0%; *P* = 0.016). Patients with LPN metastasis had inferior 3-year DFS and OS rates than those without LPN metastasis, but the differences were marginal (44.2% vs. 63.6%; *P* = 0.262 and 74.0% vs. 81.9%; *P* = 0.065, respectively) (Figure 3).

## DISCUSSION

Although lateral lymphatic flow in the rectum was confirmed in 1895 [14], controversy still remains on the treatment of metastatic LPN in locally advanced rectal cancer. In Japan, prophylactic or therapeutic LPND is routinely recommended for advanced lower rectal cancer because a considerable number of patients eventually suffer from LPN recurrences. [4, 6, 15] Contrarily, the other part of the world prefers preoperative CRT in metastatic LPN because the rate of LPN metastasis is relatively low, CRT could be as effective as surgery, and LPND causes complications without improving oncologic results, implying systemic disease. In fact, a recent study revealed that only 7.9% of advanced lower rectal cancer without evidence of LPN metastasis on preoperative imaging had LPN metastasis after prophylactic LPND. In other words, over 90% of LPND for those patients can be unnecessary overtreatment.

Standing between the both extremes, some studies have suggested selective LPND after preoperative CRT for rectal cancer patients with suspicious LPNs on imaging studies [4, 6, 16]. This would be a reasonable option, considering that we could avoid unnecessary LPND, abolish the major foci of locoregional recurrence, and consequently achieve a good selection of patients with a high LPN metastatic rate, up to 66% even after preoperative CRT [4, 6, 11].

Likewise, our strategy also used to be selective LPND, targeting only patients with persistently suspicious LPNs even after preoperative CRT. However, during the earlier study period we have found very intriguing results. Specifically, *in situ* LPN recurrence among patients in group A who had shown a good response to preoperative CRT, but had only undergone TME. Therefore, we altered the treatment policy to add LPND for those patients who then became group B later on. As a result, patients in group B had excellent oncologic outcomes in local recurrence. These clinical findings can be considered as follows.

First, *in situ* recurrence in group A is because LPNs were considered to have been completely eradicated after preoperative CRT, but the tumor burden remained within them and eventually regrew. And also this suggests that current diagnostic tools, mainly pelvic MRI, are unable to accurately detect metastatic LPNs after CRT. In this study, a short-axis diameter ≥ 5 mm was considered suspicious before CRT and non-respond after CRT, in addition to, morphologic criteria.

Valid diagnostic criteria for detecting metastatic lymph nodes on MRI are still lacking [16]. Although lymph node size is a widely accepted criterion, it has limited accuracy. This is because some small lymph nodes may be revealed metastatic and there is potential for overlap in size between metastatic and non-metastatic nodes. Several previous studies have reported that using a larger cut-off value for LPN size could reduce the false-positive rate (i.e., increasing the rate of LPN metastasis) [4, 5, 11]. Akiyoshi et al. reported that 85.7% of patients with metastatic LPNs after LPND had a short-axis diameter ≥ 8 mm before CRT [4]. The size criterion in the present study is consistent with that of a previous study (≥ 5 mm), which was a significant risk factor for pelvic recurrence in rectal cancer [10]. If the criterion for suspicious LPNs is changed from 5 to ≥ 7 mm on pretreatment MRI, the overall metastatic rate also increases from 37.7 to 44%. Additionally, a high frequency ≥ 7 mm diameters resulted in high a LPN positivity in group C (Table 1) and local recurrence only occurred in group A with suspicious LPNs with a short-axis diameter ≥ 7 mm on pretreatment MRI. However, using a larger cut-off value for LPNs can lead small-sized metastatic LPNs go undetected. Therefore, further studies are required to determine the optimal cut-off size on pretreatment MRI for detecting metastatic LPN for LPND. In contrast, the MURCCURY study group proposed morphologic criteria, such as signal heterogeneity and irregular border. However, it is difficult to reach a consensus among radiologists [17].

In respect to lymph node staging after preoperative CRT, it is difficult to adopt both the size criterion and morphologic criteria because of the downsizing effect of radiation and the absence of definitive criteria for differentiating between metastatic and irradiated lymph node change on post-CRT MRI [18]. A recent histological study reported that 95% of all nodes post-CRT were < 5 mm and that 50% of metastatic nodes were < 3 mm. This suggests that the accuracy of the nodal size criteria and morphology assessment on restaging imaging would be limited [13]. Thus, advances in post-CRT imaging studies are needed to avoid under- or overtreatment for suspicious LPNs.

Second, none of patients in group B experienced local recurrence after additional LPND were performed on responded LPNs. Similarly, five patients (16.1%) who experienced *in situ* LPN recurrence in group A only had local recurrence and, of these, 3 achieved long-term survival after the removal of recurrent LPNs by salvage LPND. Whether LPN metastasis is regional or systemic disease is also a long-standing issue in performing LPND, but a multi-center study in Japan reported that the survival rate of patients with LPN metastasis was comparable to that of patients with N2 disease. Therefore, the authors suggested that LPN metastasis could be considered a locoregional disease [19]. The Current study findings suggest that excellent local control could be achieved by preoperative CRT and additional LPND, especially in those patients who exhibited a marked response to CRT.

On contrary to good local control after LPND in groups A and B, patients in group C showed higher LPN positive rate and resulted in high local recurrence alongside poor DFS and OS rates even after LPND. However, poor oncologic outcome in group C did not seem to be caused by only metastatic LPNs. Patients in group C showed the poor response to preoperative CRT, and consequently, advanced pathologic tumor and node staging with higher LPN metastasis were observed in these patients. Some previous studies have suggested that patients with positive pelvic nodes had inferior DFS and OS compared to those without positive nodes, even after LPND [20–22]. In contrast, another previous study, after controlling for confounding factors, suggested that LPN metastasis itself after preoperative CRT and additional LPND was not a poor prognostic indicator [4]. Our results were close to a later study [4]. The higher local recurrence in group C could be attributed to either incomplete LPND or possible tumor cell spillage during extensive LPND. In addition, the poor response to preoperative CRT in group C could be attributed to the biology of the tumor, which might be more aggressive than others. Thus, in groups C, neither CRT nor LPND could effectively control the disease. However, it is clear that the degree of response to CRT of suspicious LPNs can be an early response indicator in patients with rectal cancer, which can be used to predict oncologic outcomes. Therefore, in these patients, advanced treatments are required such as preoperative CRT with active agents or consolidation chemotherapy to improve the response to CRT or adjuvant advanced chemotherapy after radical resection.

Preoperative CRT can reduce tumor volume, sterilize lymph node, and improve local control. In this study, 49 (58.3%) of 84 patients were diagnosed as having threatened or invaded mesorectal fascia on pretreatment MRI. After preoperative CRT, 6 patients (7.1%) revealed circumferential resection margin positive on final pathologic examination. Of these 3 patients developed central pelvic recurrence. Preoperative CRT can also sterilize mesorectal or pelvic lymph nodes. However, in this study, tumor burden was remained in 16.7% of patients in group B, even though they exhibited a good response to preoperative CRT, and 65.2% of patients in group C. It may be immoderate to suggest an indication for LPND after preoperative CRT. However, based on the findings of this study, such as *in situ* LPN recurrence in group A, the excellent local control in group B, and the diagnostic insufficiency of pelvic MRI for LPNs metastases after CRT, TME alone is not sufficient in treating suspicious LPNs detected on pretreatment imaging. Furthermore, we may suggest that if the short-axis diameter is ≥ 7 mm it is favorable to perform LPND, even when a good response to preoperative CRT is observed. A previous study that found lateral pelvic recurrence in 70.8% of rectal cancer patients with suspicious LPNs on pretreatment pelvic MRI and who were treated by preoperative CRT only, but were not surgically removed, also supported our strategy for LPND [10].

Our latest policy tends toward more active application of LPND based on preoperative imaging diagnosis, rather than response to preoperative CRT. To put in practice of this policy was possible due to advances in surgical technique and imaging study. Early experiences of LPND have resulted in high morbidity and severe urinary and sexual dysfunction, increasing more than three times compared to conventional TME [13, 23]. However, recent studies have reported that with the application of pelvic autonomic nerve-preserving techniques, LPND does not increase the risk of postoperative complications and functional impairments [24, 25]. Furthermore, recent advances in instruments and magnified imaging system in minimally invasive approach enable the surgeon to perform LPND with lower complication rates. However, detailed comparison of functional outcomes between TME and additional LPND are required [13, 24].

This study had several limitations. First, it was a retrospective study; therefore, there could be selection bias. Second, the number of patients was relatively small, and the follow-up period for some patients was not long enough to evaluate the recurrence adequately. Third, our policy in treating LPNs in rectal cancer changed during study periods, which may also lead to selection bias. However, this change provided the opportunity to investigate the outcomes of performing LPND in relation to response to preoperative CRT and provided reference of treatment strategies in patients with suspicious LPNs. However, these results should be confirmed by large-cohort studies with long-term follow-up in rectal cancer patients who have suspicious LPNs.

In the present study, we experienced an unexpectedly large proportion of *in situ* recurrences originating from tumors primarily located in the lateral pelvic side-wall. Findings suggest that LPND cannot be omitted, regardless of the response to preoperative CRT, in patients with suspicious LPN on pretreatment imaging owing to higher local recurrence rates. In addition, LPND for responded LPNs achieved good local control. However, more intensive treatments are required to improve survival in patients without clinical response to preoperative CRT on LPN even after additional LPND because it can be an early predictor of oncologic outcomes in locally advanced rectal cancer. Furthermore, the establishment of a method with increased diagnostic capability to detect metastatic LPNs would be of major interest for patients with rectal cancer, both to guide surgery, thus allowing LPND, and to guide pathologic analysis.

## MATERIALS AND METHODS

Between January 2006 and December 2013, 402 patients with locally advanced rectal cancer underwent preoperative CRT, followed by TME. Twenty-five patients were excluded from this study by the reasons including distant metastasis, local excision, history of another malignancy, and absence of MRI findings. Patients’ demographic characteristics, imaging studies, operative and pathologic findings, and follow-up data were reviewed retrospectively from a prospectively collected computerized database of patients with colorectal cancer.

Long-course preoperative CRT was administered to patients with clinical T3, T4, or node-positive disease who had mid to lower rectal cancer. Radiation was administered to the whole pelvis at a dose of 45 or 50 Gy in 25 fractions over 5 weeks. Chemotherapy was based on 5-fluorouracil, either as a bolus infusion (425 mg/m^2^/day) in combination with leucovorin (20 mg/m^2^/day) or as a continuous infusion for 5 days (250 mg/m^2^/day) during the first and fifth weeks of radiotherapy. Curative radical resection was performed 6–8 weeks after the completion of radiotherapy.

Suspicious LPN was defined as a short-axis diameter of ≥ 5 mm with speculated or indistinct borders, or a mottled heterogenic pattern on MRI, or “hot-uptake” on PET/CT. After completing radiotherapy, the clinical response of the pelvic nodes was reexamined by pelvic MRI within 7 days before surgery with the same protocol at initial study. In this study, responded LPN was defined as a decrease in the short-axis diameter to < 5 mm. The decision to conduct LPND was determined in a multidisciplinary meeting that included a surgeon, an oncologist, and a radiologist. Until 2010, LPND adding to TME was mainly performed in patients with persistently suspicious LPNs after preoperative CRT. However, after 2011, LPND was performed in all patients with suspected metastatic pelvic lymph nodes on pretreatment imaging, irrespective of the clinical response shown by post-CRT MRI.

The surgical technique for TME with LPND has been described previously [20]. Dissection of the lateral nodes outside of the pelvic plexus was considered to be performed in six zones: the internal iliac, mid-rectal, obturator, common iliac, external iliac, and aortic bifurcation areas [14, 26]. Generally, lymphadenectomy outside of the external iliac vessels and in the para-aortic area are not performed, except in patients with highly suspicious metastatic nodes along those vessels. When metastatic lymph nodes were found or suspected in the lateral lymphatic channels around the internal iliac vessels or its branches, the vessels were preserved to the extent possible. However, when metastatic lymph nodes encapsulated these structures, *en bloc* resection of these vessels was aggressively carried out.

Local recurrence was defined as tumor recurrence within the pelvic cavity, and distant metastasis was defined as any recurrence outside of the pelvic cavity. Pelvic side-wall recurrence was defined as recurrence in the LPN-bearing areas, outside the mesorectal fascia along the obturator, internal, and external iliac vessels.

Continuous data are presented as means (s.d.). Between-group differences in continuous data were analyzed using the Student’s *t* test and Mann-Whitney *U* test for independent values for normally and non-normally distributed values, respectively. Categorical data were compared using χ^2^ test or Fisher’s extract test, as appropriate. Groups were compared on an intention-to-treat basis. Disease-free survival (DFS) and overall survival (OS) were estimated using the Kaplan-Meier method. A two-sided *P* value of < 0.05 was considered statistically significant. All statistical analyses were performed using SPSS software version 17.0 (SPSS Inc, Chicago, IL, USA).
